# Pathogenicity evaluation of GVI-1 lineage infectious bronchitis virus and its long-term effects on reproductive system development in SPF hens

**DOI:** 10.3389/fmicb.2022.1049287

**Published:** 2022-10-28

**Authors:** Zongyi Bo, Shuqin Chen, Chengcheng Zhang, Menjiao Guo, Yongzhong Cao, Xiaorong Zhang, Yantao Wu

**Affiliations:** ^1^College of Veterinary Medicine, Jiangsu Co-Innovation Center for the Prevention and Control of Important Animal Infectious Disease and Zoonoses, Yangzhou University, Yangzhou, Jiangsu, China; ^2^Joint International Research Laboratory of Agriculture and Agri-Product Safety, The Ministry of Education of China, Yangzhou University, Yangzhou, China

**Keywords:** infectious bronchitis virus, GVI-1 lineage, pathogenicity, early infection, reproductive system, egg production

## Abstract

Infectious bronchitis virus (IBV) has gained increasing attention in the poultry industry due to its ability to cause tissue injuries not only in the respiratory system and kidney but also in the reproductive system of layers. Recently, the GVI-1 lineage IBVs have spread widely in China, whereas their pathogenicity in egg-laying chickens has rarely been studied, especially its long-term influence in egg production upon the early infection in chicks. In this study, 10-day-old SPF chicks were infected with the GVI-1 lineage JX181 strain and monitored over a 170-day period after infection. The pathogenicity evaluation of the JX181 strain included clinical observations, immunohistochemical assay, viral load, viral shedding, gross autopsy, and laying rate. The results showed that JX181 has a high pathogenicity, causing severe system lesions, and the decrease in egg production. In summary, this study describes the long-term damages caused by the early infection with the IBV GVI-1 lineage on the reproductive system of hens, providing a comprehensive understanding of the pathogenicity of the IBV GVI-1 lineage and emphasizing the importance of its early prevention.

## Introduction

Infectious bronchitis virus (IBV), the causative agent of avian infectious bronchitis, has been widely prevalent worldwide since it was first reported in the 1930s (Beach and Schalm, 1936). IBV infects chickens of all ages and breeds, resulting in respiratory tract damage, nephritis, and reproductive problems, such as declines in egg production and quality (Cavanagh, 2007). IBV belongs to the gamma-coronaviruses, and its genome is a single positive-strand RNA with a high mutation and recombination rate (Thor et al., 2011), which leads to the continuous emergence of new genotypes and serotypes (Jackwood, 2012). To date, eight genotypes (GI ~ GVII) and more than 30 distinct viral lineages of IBV have been defined worldwide based on the complete S1 gene sequences (Valastro et al., 2016; Chen et al., 2017; Ma et al., 2019; Domanska-Blicharz et al., 2020).

As the most effective method to prevent and control infectious bronchitis, vaccination is widely used in the poultry industry. Nonetheless, outbreaks of IBV continue to occur, and multiple IBV genotypes are cocirculating in China (Li et al., 2010; Zhang et al., 2021). The GI-19 (QX) genotype first appeared in the 1990s and was associated predominantly with proventriculitis, respiratory stress, nephritis, and false layer syndrome (Liu and Kong, 2004; Fan et al., 2019). The proportion of GI-19 isolates has increased continuously over the past two decades and has become the most frequently isolated IBV genotype in China at present (Zhao et al., 2016). In addition, the GI-7 (TWI) lineage has been the second most prevalent type since the first report in the Chinese mainland in 2009, which showed similar pathogenicity to strains of the QX genotype (Xu et al., 2018; Zhang et al., 2020). The GVI-1 lineage TC07-2 strain was first isolated in Guangdong, China, in 2007 and subsequently occurred in many other countries in Asia (Mase et al., 2010; Lim et al., 2012; Raja et al., 2020). Epidemiological surveillance data demonstrated that the detection rate of the GVI-1 strains has continually increased in recent years, especially in southern China (Fan et al., 2019; Chen et al., 2021). The existing results have revealed that GVI-1 strains show a high affinity for the respiratory tract rather than the kidney (Jang et al., 2018; Sun et al., 2021). However, there are few systematic studies on the pathogenicity of IBV GVI-1 strains, especially the effects of its early infection in chicks to the long-term performance of laying hens.

The objective of this study was to comprehensively evaluate the pathogenicity of the GVI-1 lineage IBV by examining clinical signs, gross lesions, histological lesions, viral shedding, and egg production in infected chickens. This research revealed that early infection with the GVI-1 lineage JX181 strain induced severe lesions in different organs, which finally resulted in reduced egg production.

## Materials and methods

### SPF chickens and embryonated chicken eggs

SPF white leghorn chickens were purchased from Jinan Sipai Furui Livestock Technology Co., Ltd. (Jinan, China). SPF embryonated chicken eggs were purchased from Beijing Boehringer Ingelheim Merial Vital Laboratory Animal Technology Co., Ltd. (Beijing, China).

### Virus isolation and titration

The IBV GVI-1 lineage strain CK/CH/JX/2018/1 (abbreviation: JX181) was isolated from a 200-day-old parent egg breeder chicken flock that showed respiratory signs, diarrhea, and egg production dropping in 2018 in Jiangxi Province of China. The virus was propagated in embryonated SPF eggs. The allantoic fluid was harvested and titrated by inoculating serial 10-fold dilutions of the virus into 10-day-old SPF embryos. The 50% embryo infectious doses (EID_50_) were determined as described by Reed and Muench (Reed and Muench, 1938).

### Pathogenicity evaluation of the JX181 strain in SPF chickens

A total of 70 female SPF chicks were randomly divided into two groups of 35 chicks each. Ten-day-old chicks in the challenge group were inoculated with 100 μl of PBS containing 10^6.5^ EID_50_ of the JX181 strain *via* the ocular-nasal route, and those in the control group were inoculated with equal amounts of PBS. Throughout the study period of 170 days, the chickens in the two groups were raised in two separate negative-pressure isolators under uniform standard management conditions with feed and water provided *ad libitum*.

Clinical symptoms (dyspnea, tracheal rales, depression, anorexia, and diarrhea) were monitored daily after the virus challenge. Three chicks from each group were selected randomly and euthanized by cervical dislocation at 3, 6, 9, 12, and 15 dpi. Necropsies were performed to observe gross lesions, and tissue samples from the trachea, lung, spleen, kidney, and bursa of Fabricius were collected individually to determine viral load by quantitative reverse transcriptase PCR (RT–qPCR) or placed into 10% neutral-buffered formalin for histologic evaluation. Oral and cloacal swabs were collected from 10 chicks in the challenge and control groups at 3, 6, 9, 12, and 15 dpi and kept in separate tubes containing 500 μl of sterile PBS to detect viral shedding.

The remaining 20 hens from each group continued to be fed in negative pressure isolators, and their reproductive performance was observed. The number of eggs and the egg quality parameters, such as albumen height, egg shape index, and eggshell thickness, were recorded daily after egg laying as described previously (Wang et al., 2014). All hens were euthanized by cervical dislocation at 170 dpi and necropsied. The length of the oviducts was measured, and the number of ovarian follicles with a diameter larger than 10 mm was recorded to evaluate the development of the reproductive system (Robinson and Etches, 1986; Zhang et al., 2020).

### Real-time quantitative PCR

Total RNA of the tissues (trachea, lung, spleen, kidney, and bursa of Fabricius) and oral/cloacal swabs were extracted with the Ultrapure RNA Kit (CoWin Biosciences, Beijing, China) according to the manufacturer’s instructions. The cDNA was prepared by reverse transcription using the EasyScript® Reverse Transcriptase [M-MLV, RNaseH-] Kit (TransGen Biotech, Beijing, China). Previously described primers and probe (Callison et al., 2006) were used for RT–qPCR, which was performed using LineGene 9,600 Plus (FQD-96A, Bioer Technology, Hangzhou, China). The reaction mixture comprised 10 μl of AceQ qPCR Probe Master Mix (Vazyme Biotech Nanjing, China), 0.4 μl of forward primer, 0.4 μl of reverse primer, 0.2 μl of probe and 2 μl of cDNA (or nuclease-free water for the control). ddH_2_O was added to a total volume of 20 μl. The thermal cycling parameters were as follows: 95°C for 5 min, followed by 40 cycles of 95°C for 10 s and 60°C for 30 s.

### Histopathology

Tissue samples from the trachea, lung, spleen, kidney, and bursa of Fabricius were collected and fixed in 10% neutral-buffered formalin for 48 h and embedded in paraffin wax. Sections (5 μm thick) were cut and stained with hematoxylin and eosin (H&E) to examine pathological changes in tissues under a microscope.

### Statistical analysis

All statistical analyses were performed using GraphPad Prism 8.0.2 (San Diego, CA, United States). Statistical differences between two groups were assessed by the Student’s *t*-test Statistical significance was defined as follows: *p < 0.05, **p < 0.01, ***p < 0.001.

## Results

### Severe clinical signs and gross lesions were shown in JX181 challenged chicks

**Figure 1:**
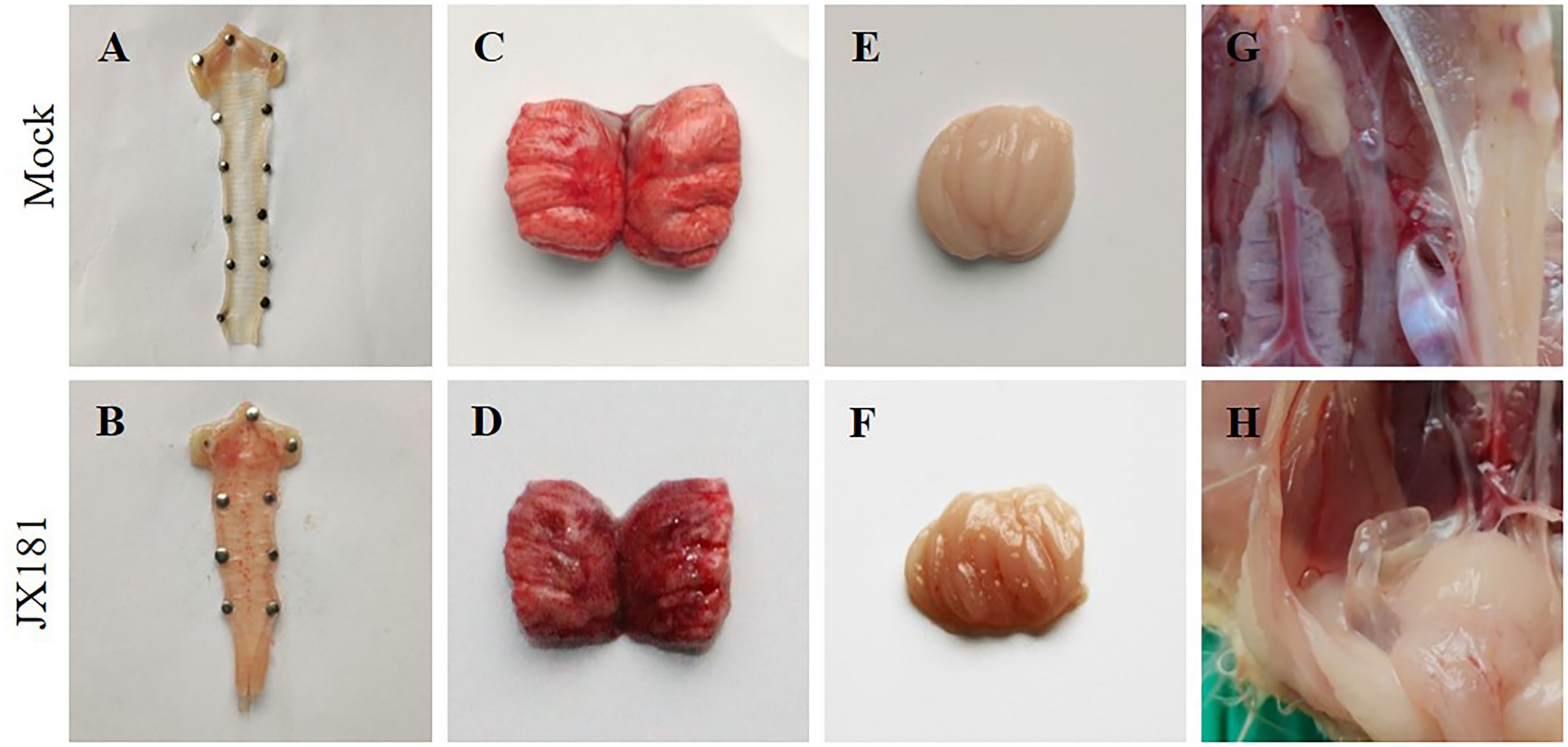
Gross lesions in the different organs of chicks inoculated with strain JX181. 10-day-old chicks were challenged with 10^6.5^ EID_50_ of JX181 *via* the ocular–nasal route. The representative gross lesions of the chicks sacrificed at 6 dpi were shown in **(A,B)** Larynx and trachea, **(C,D)** Pulmonary congestion, **(E,F)** Bursa of Fabricius, **(G,H)** Oviduct.

To explore the long-term pathogenicity of the GVI-1 lineage to chickens, 10-day-old chicks were challenged with 10^6.5^ EID_50_ of JX181 *via* the ocular–nasal route. The results demonstrate that the morbidity of the early infection of IBV GVI-1 lineage JX181 strain was 100%, the mortality was 0%, and early infection with JX181 strain could cause obvious clinical signs in chicks from 3 to 12 dpi, which primarily included the tracheal rales, dyspnea, depression, and ruffled feathers. Compared with the control group, necropsy revealed that the challenged chicks showed catarrhal exudate and severe punctate hemorrhage in the larynx and trachea (Figures 1A,B), pulmonary congestion (Figures 1C,D), hemorrhage in the bursa of Fabricius (Figures 1E,F), and cystic dilations in the oviduct (Figures 1G,H) from 3 to 12 dpi. No obvious renal lesions were found in challenged chicks.

### Histopathological lesions were induced upon JX181 challenge

**Figure 2:**
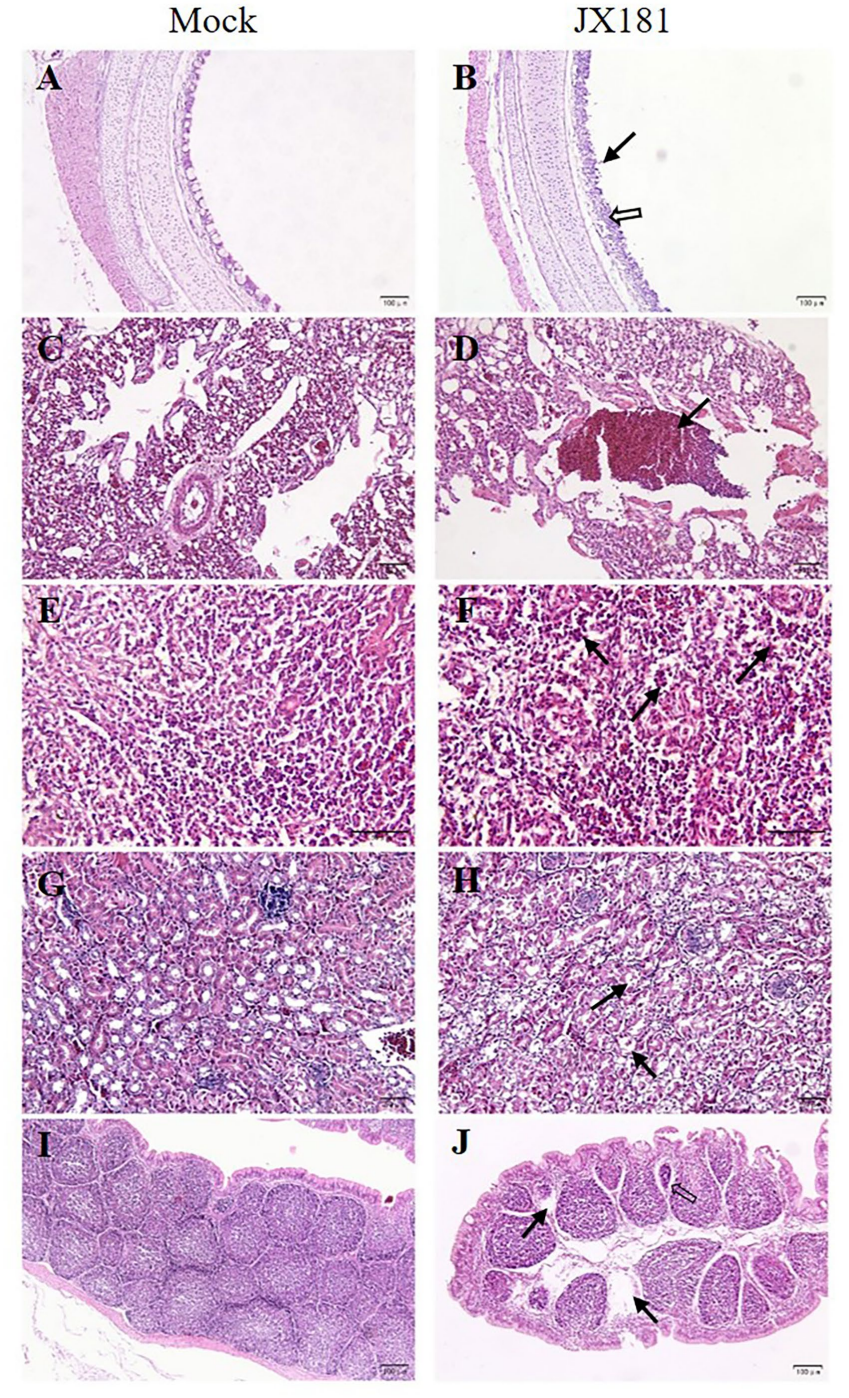
Histopathologic changes were observed in different tissues of chicks inoculated with strain JX181 at 6 dpi. **(A,B)** Trachea: the black arrow indicates the extensive loss and necrosis of ciliated epithelial cells, and the hollow arrow indicates the thickening of the lamina propria of the tracheal mucosa and lymphocyte infiltration. **(C,D)** Lung: the black arrow indicates erythrocyte infiltration in the bronchial lumen. **(E,F)** Spleen: black arrows indicate macrophages in the splenic sinus. **(G,H)** Kidney: black arrows indicate swelling and degeneration of tubular epithelial cells. **(I,J)** Bursa of Fabricius: the hollow arrow indicates the interstitial dilation of the lymphoid follicles, and black arrows indicate the lymphocyte loss.

Histopathological examination was performed to check whether JX181 infection could induce histopathological lesions. The results showed noticeable pathological changes in the tissues of challenged chicks, which were predominant at 6 and 9 dpi. Compared with the control group, cilia loss, sloughing of epithelial cells, thickening of the lamina propria of the tracheal mucosa, and lymphocyte infiltration in the tracheas of chicks were observed in the challenge group (Figures 2A,B). Bronchial hemorrhage was seen in the lungs, and there was extensive erythrocyte infiltration in the lumen (Figures 2C,D). In the spleen, the number of macrophages in the splenic sinus was increased (Figures 2E,F). In the kidney, the tubular epithelial cells exhibited swelling and vacuolar degeneration (Figures 2G,H). In the bursa of Fabricius, interstitial dilation of the lymphoid follicles, atrophy of lymph follicles, and lymphocyte loss were observed (Figures 2I,J).

### The viral loads and viral shedding remained positive early after incubation

**Figure 3:**
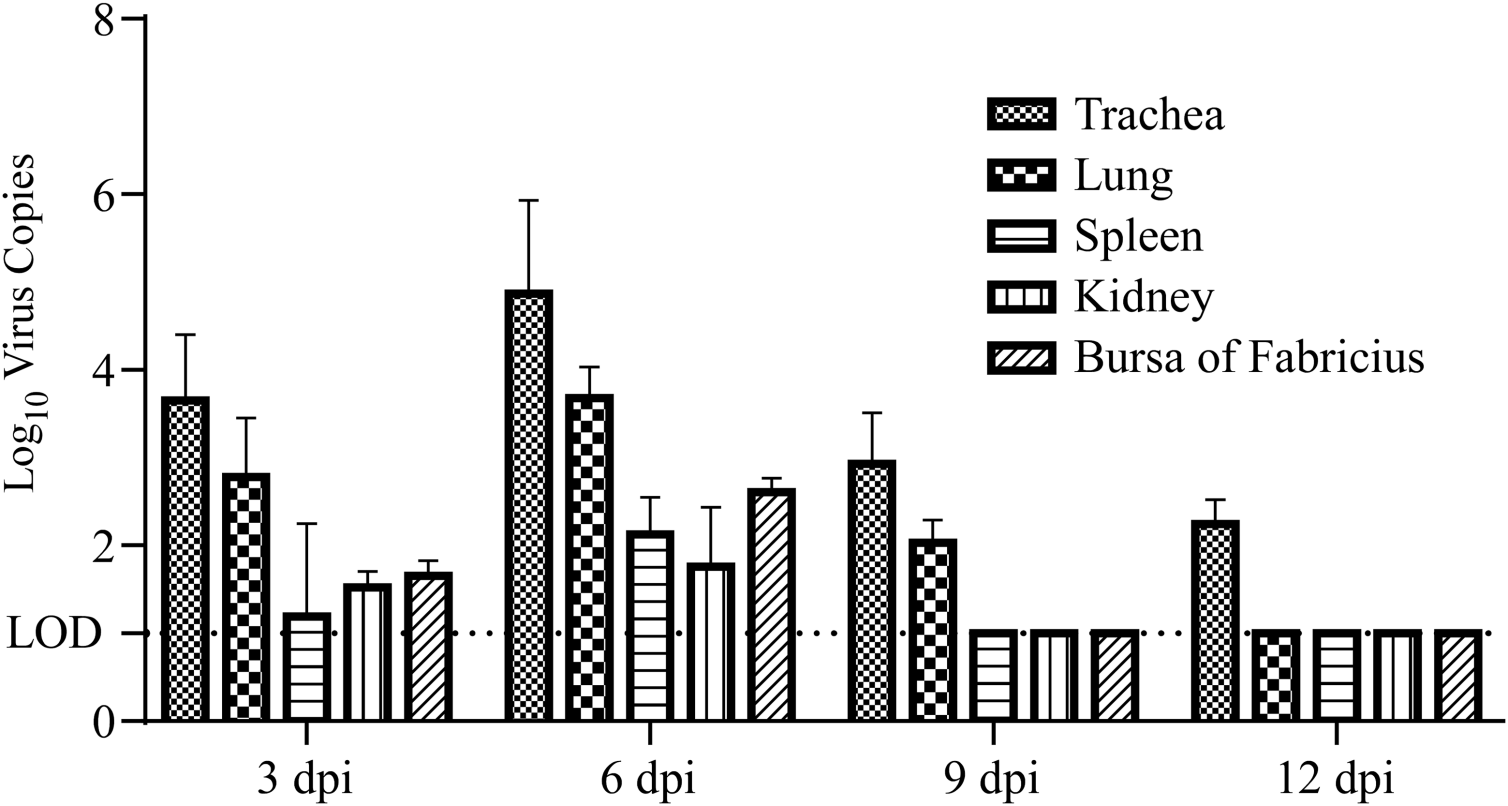
Viral load in different tissues of chickens at 3, 6, 9, and 12 days post infection. The trachea, lung, spleen, kidney, and bursa of Fabricius samples were collected at 3, 6, 9, and 12 dpi, total RNA was extracted and reverse transcribed into cDNA, and RT–qPCR was used to measure the viral load in different tissues. LOD: limit of detection.

**Figure 4:**
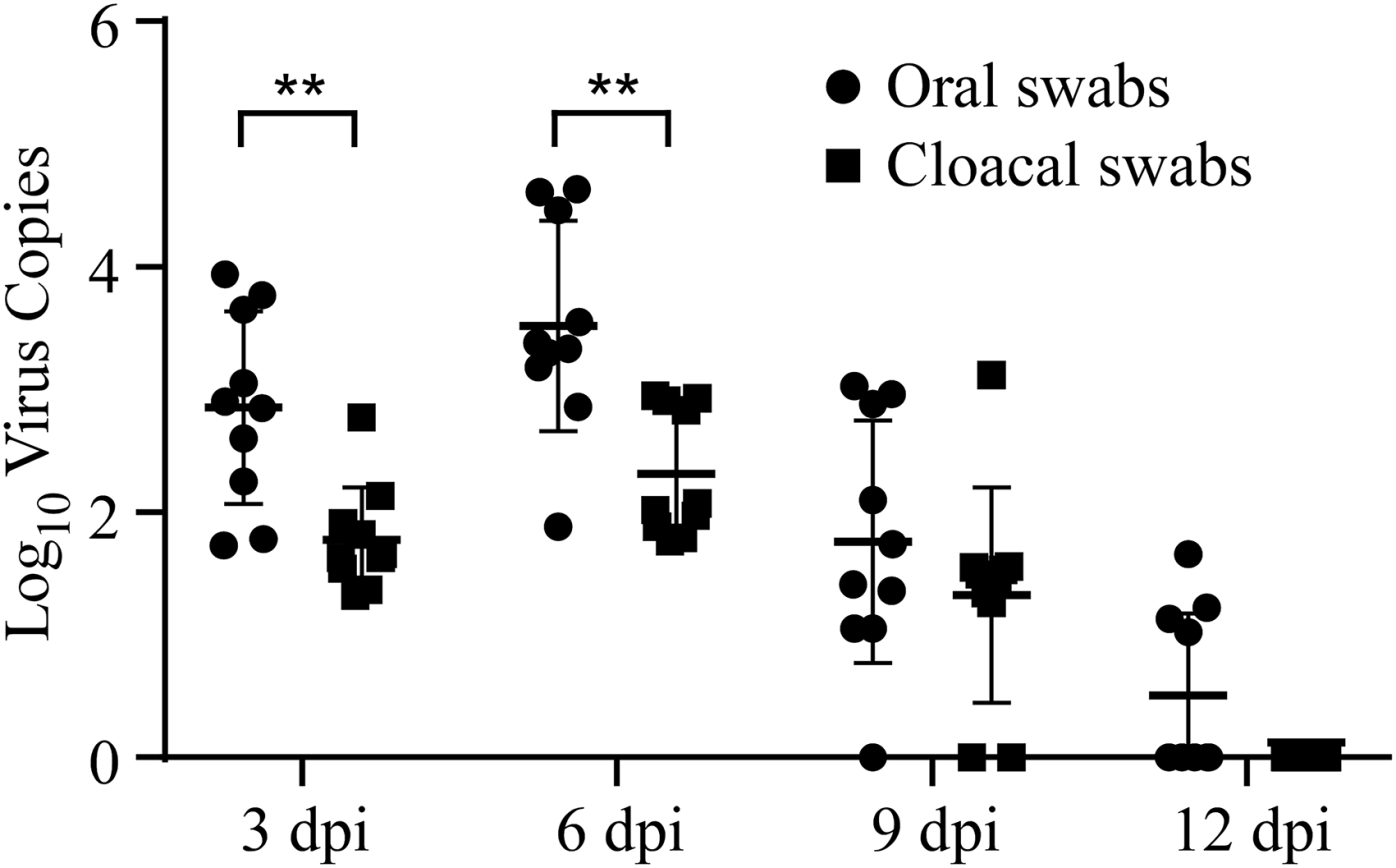
Viral shedding was detected in both oral and cloacal swab samples. Oral and cloacal swab samples were collected at 3, 6, 9, and 12 dpi, and RT-qPCR was used to measure the level of viral shedding in these samples. Statistical differences between two groups were assessed by the Student’s *t*-test Statistical significance was defined as follows: **p < 0.01.

Viral loads and shedding were detected to measure the dynamics of the virus in the challenged chicks. First, RT–qPCR was used to detect the dynamics of viral loads in different tissues of the sacrificed challenged chicks, including the trachea, lung, spleen, kidney, and bursa of Fabricius. The results demonstrated that the dynamics of virus copies in different kinds of collected tissues showed similar trends, which increased from 3 dpi to 6 dpi, peaked at 6 dpi, and then subsequently declined after 6 dpi (Figure 3). At 12 dpi, IBV could only be detected in the trachea, and it could not be further detected at 15 dpi (Figure 3). No viral RNA in tissues was detected at any time in the control group. Second, oral and cloacal swabs were collected from 10 chicks in the challenge group and control group at 3, 6, 9, 12, and 15 dpi. Similar to the viral load results, IBV viral shedding peaked at 6 dpi and then decreased in both oral and cloacal swabs. No viral shedding was detected in cloacal swabs, while 40% (4/10) of oral swabs were positive at 12 dpi (Figure 4). No viral shedding was detected in oral and cloacal swabs at 15 dpi. The results demonstrated that viral shedding in the oral swabs was slightly higher than that in the cloacal swabs. Taken together, these data demonstrate that the viral load and shedding could be detected for the first 12 days after the challenge.

### Early infection with IBV JX181 resulted in a lower laying rate

**Figure 5:**
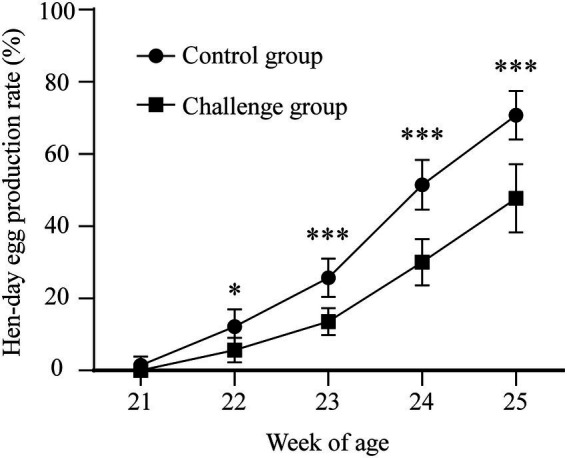
Decreased egg production was observed in the challenge group. The number of eggs in each group was counted from 21 to 25 weeks. Statistical differences between two groups were assessed by the Student’s *t*-test Statistical significance was defined as follows: *p < 0.05, ***p < 0.001.

The hen laying rate was measured to check whether early IBV infection in chicks could affect later egg production. The number of eggs in each group was counted, and the laying rate was measured. The results showed that the egg production of the challenge group was obviously lower than that of the control group from 21 to 25 weeks (Figure 5). However, there were no significant differences between the two groups in the egg shape indices and eggshell thickness. These data demonstrated that although viral loads and shedding of JX181 were positive for no more than 15 days, the performance of egg laying was severely affected by JX181 infection.

### Reproductive system lesions were the causative factor of the decreased laying rate

**Figure 6:**
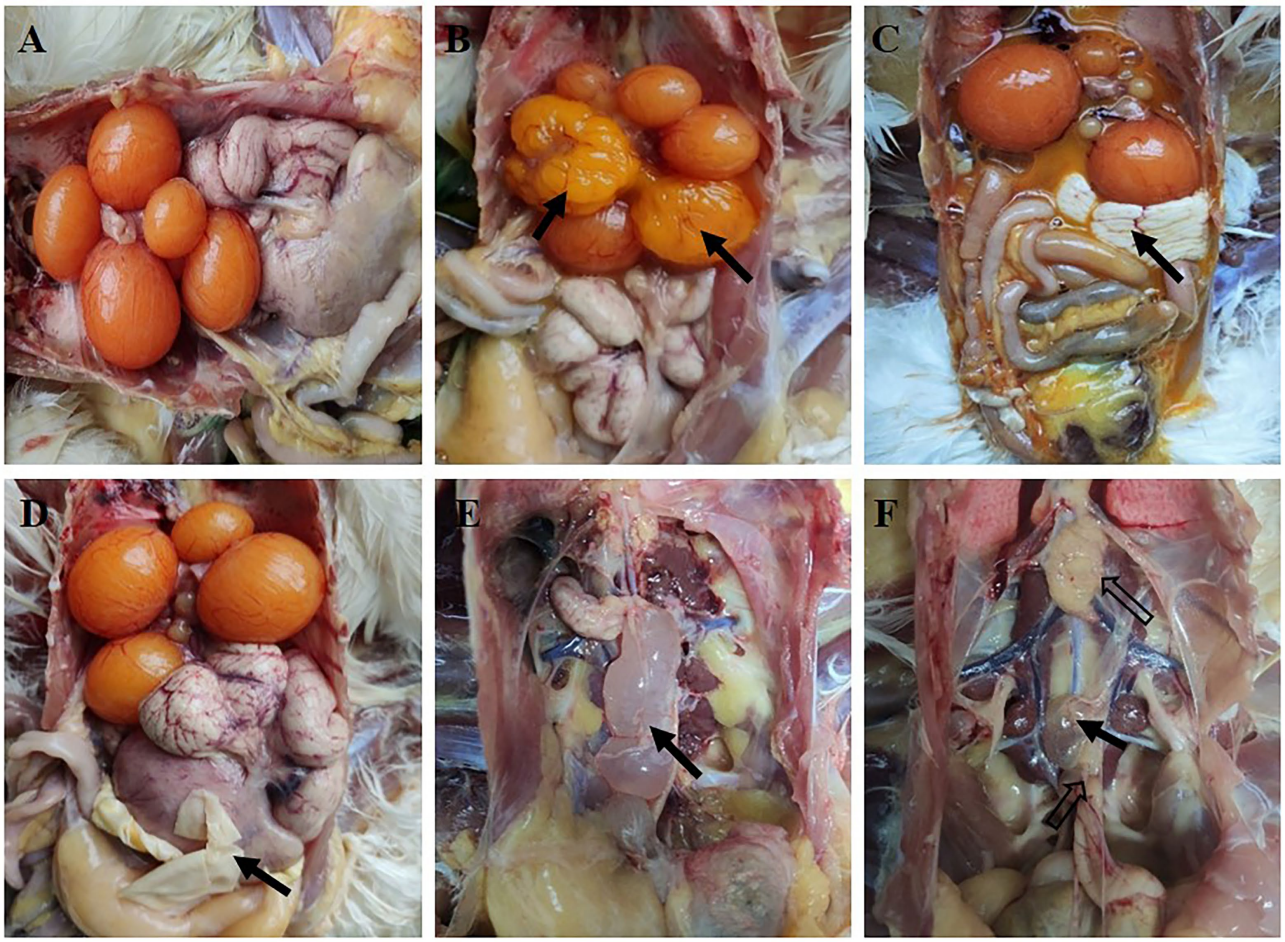
Gross lesions were observed in the ovary and oviduct of the hens at 170 dpi. **(A)** Well-developed organs from the control group. **(B)** Flaccid ovary. **(C,D)** The fluid yolk material and fibrin clots in the coelome. **(E)** Cystic dilation in the oviduct. **(F)** Oviduct and ovary with retarded development.

**Figure 7:**
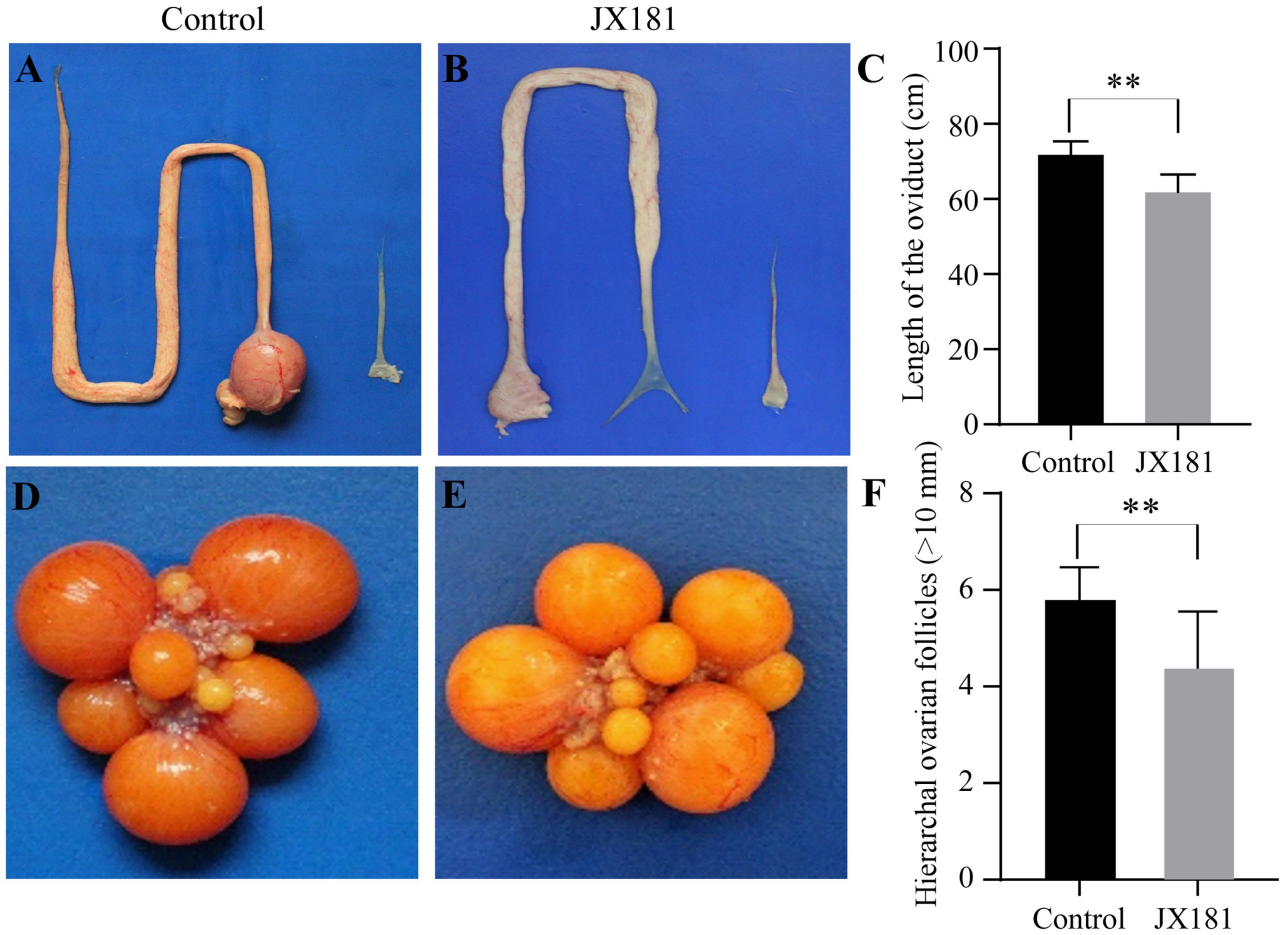
The lengths of the oviducts and the number of hierarchal ovarian follicles were impaired upon challenge with JX181 at 170 dpi. **(A,B)** The lengths of the oviducts were measured after the euthanasia of the chickens. **(C)** Statistical representation of the mean lengths of the oviducts. **(D,E)** The hierarchal ovarian follicles in mock and JX181 infected groups. **(F)** Statistical representation of the number of hierarchal ovarian follicles with diameters larger than 10 mm.

As the above data showed that the laying rate was decreased after early infection with JX181, we proposed that reproductive system lesions might contribute to this influence. All hens were euthanized by cervical dislocation at 170 dpi, and the necropsy results showed that the reproductive system of hens in the control group was well developed without any lesions (Figure 6A), while various types of lesions were observed in oviducts and ovaries in the challenge group. Specifically, 2/20 of the hens showed degenerated ovarian follicles (Figure 6B). Free yolk or fibrin clots were observed in the abdomen in 6/20 of the hens (Figures 6C,D). Sixteen hens in the challenge group had varied in size cystic dilatations with a watery content in the oviduct (Figure 6E). The development of oviducts and ovaries was also assessed, and 5/20 of hens showed moderately to severely retarded development of oviducts and ovaries (Figure 6F). Moreover, the statistical analysis of the lengths of the oviducts (Figures 7A–C) and the number of hierarchal ovarian follicles with diameters larger than 10 mm (Figures 7D–F) were decreased upon JX181 infection. Collectively, these data demonstrated that early infection of chicks with the IBV GVI-1 lineage JX181 strain has a long-term effect on the development of the layer reproductive system.

## Discussion

IBV replicates in various organs, including the respiratory and intestinal tract, kidney, oviduct, and testes (Bande et al., 2016). Different genotypes of IBV exhibit varying virulence and tropism and cause different clinical symptoms. In recent years, the frequency of isolation of the GVI-1 lineage from vaccinated flocks has increased. Several previous studies have proven that GVI-1 viruses have strong respiratory tropism, but there are few studies on the pathogenicity of those strains in the reproductive system (Fan et al., 2019; Ma et al., 2019; Ren et al., 2019; Sun et al., 2021).

IBV infection may lead to two different forms of hen reproductive system disease (Hoerr, 2021). If the infection occurs during laying, it may lead to transiently decreased egg production accompanied by eggshell deformities and deterioration of egg quality. If the infection occurs in naïve pullets, it can lead to false layer syndrome, which is characterized as a mature hen with active ovaries but severe cystic dilation of the oviduct. The JX181 strain was first isolated from layers with decreased egg production. Therefore, we speculate that if infection occurs in early stages after hatching, the virus may also cause false layer syndrome. If this is the case, prevention and control of the loss caused by GVI-1 strains will be extremely difficult due to the large antigenicity difference between the GVI lineage strains and the currently commonly used vaccine strains, such as H120, Ma5, and 4/91 (Ma et al., 2019).

In the present study, we modeled GVI-1 JX181 infection in 10-day-old SPF chicks with long-term monitoring and explored its pathogenic characteristics. This result revealed that the GVI-1 lineage JX181 strain had a short incubation period and induced severe respiratory symptoms in the initial stage of infection. The autopsy results also indicated that JX181 could cause serious hemorrhage in the trachea and pulmonary congestion. These results were consistent with other studies on the pathogenicity of the GVI strain, which reflected the nature of the pathogenicity of GVI virus to the chicken respiratory system (Wang et al., 2022). However, in this study, the JX181 strain showed only moderate renal pathogenicity. Although the infected chickens showed some histological changes in the kidney at 6 and 9 dpi, there were no obvious gross lesions in it. We also noticed that although the incidence of the JX181 strain in the challenge group reached 100%, no chicken deaths occurred, indicating that JX181 was relatively mild compared with the other two genotypes (QX and TWI) that are currently widely circulating in China. Our analysis suggests that this low mortality may be related to the low renal pathogenicity of this strain because some studies have shown that the renal pathogenicity of IBV is one of the important reasons for the increased mortality due to kidney failure in susceptible birds (Chong and Apostolov, 1982; Butcher et al., 1990). Moreover, the JX181 strain also showed tropism for immune organs, including the spleen and bursa of Fabricius. Whether the ability of viruses to infect the immune system will lead to host immunosuppression still needs further study.

The incidence rate of oviduct damage has been reported to decrease with increasing age of exposure (Broadfoot et al., 1956). The pathogenic role of IBV on the oviduct differed strongly across strains. Five QX-like IBV strains can cause dilatation and serous fluid accumulation in the oviduct in different proportions but not in the 793/B strain (Benyeda et al., 2009). In our previous study, the second most prevalent genotype, TW I (also designated the GI-7 lineage), was demonstrated to also induce severe cystic oviduct in challenged chickens (Zhang et al., 2020). In the present study, cysts in the oviduct could be observed in approximately 80% of hens after infection with the JX181 strain, and this lesion was first found as early as 6 dpi and could still be observed at the end of the experiment at 170 dpi. These results indicate that GVI-1 IBV can cause irreversible damage to the oviducts of hens. The liquid-like yolk material and fibrin clots in the body cavity of hens are likely the result of oviduct damage resulting in dysfunction of actively capturing ova. A Korean study showed that K-I genotype virus infection inhibited the formation of hierarchal ovarian follicles in 80% and oviduct maturation in 50% (Hong et al., 2012). Likewise, we also observed a decrease in the number of hierarchal ovarian follicles, and the reproductive system of hens had stunted growth after infection with GVI-1 JX181 strain. Additionally, the length of the oviduct and the number of ovarian follicles in the challenge group decreased compared with those in the control group. All of these lesions in the reproductive system induced by early infection with the JX181 strain resulted in an obvious decrease in egg production.

In conclusion, this study showed that early infection with the IBV GVI-1 lineage JX181 strain is highly pathogenic to chickens and induces serious respiratory injuries, permanent damage to the oviduct, and reproductive system growth retardation, which ultimately results in a decrease in egg production. Our study comprehensively revealed the long-term influence of IBV infection, which proved that the early infection of IBV could lead to a decreased laying rate.

## Data availability statement

The original contributions presented in the study are included in the article/supplementary material, further inquiries can be directed to the corresponding authors.

## Ethics statement

This study was approved by the Experimental Animal Ethics Committee of Yangzhou University (approval ID: YZUDWLL-201906-008). The experimental design met the requirements of ethical principles related to animal experiments.

## Author contributions

XZ, ZB, and SC conceived and designed the experiments. SC performed the experiments. XZ, SC, MG, CZ, and YC analyzed the data. SC and ZB wrote the paper. XZ and YW read and approved the manuscript. All authors contributed to the article and approved the submitted version.

## Funding

This study was supported by the National Natural Science Foundation of China (31872496), the China Agriculture Research System of MOF and MARA (CARS-40), the Key Special Project “Science and Technology Promote Economy of 2020” of the National Key Research and Development Program (SQ2020YFF0426460), and the Priority Academic Program Development of Jiangsu Higher Education Institutions (PAPD).

## Conflict of interest

The authors declare that the research was conducted in the absence of any commercial or financial relationships that could be construed as a potential conflict of interest.

## Publisher’s note

All claims expressed in this article are solely those of the authors and do not necessarily represent those of their affiliated organizations, or those of the publisher, the editors and the reviewers. Any product that may be evaluated in this article, or claim that may be made by its manufacturer, is not guaranteed or endorsed by the publisher.
